# Gallotannin-Enriched Extract Isolated from Galla Rhois May Be a Functional Candidate with Laxative Effects for Treatment of Loperamide-Induced Constipation of SD Rats

**DOI:** 10.1371/journal.pone.0161144

**Published:** 2016-09-12

**Authors:** Ji Eun Kim, Jun Go, Eun Kyoung Koh, Sung Hwa Song, Ji Eun Sung, Hyun Ah Lee, Young Hee Lee, Jin Tae Hong, Dae Youn Hwang

**Affiliations:** 1 Department of Biomaterials Science, College of Natural Resources & Life Science/Life and Industry Convergence Research Institute, Pusan National University, Miryang, Korea; 2 Department of Organic Materials Science and Engineering, Pusan National University, Busan, Korea; 3 College of Pharmacy, Chungbuk National University, Chungju, Korea; Digestive Disease Research Center, Scott & White Healthcare, UNITED STATES

## Abstract

Several natural products containing tannins are used as traditional medicines for treatment of constipation; however, their pharmacological mechanism is not well understood. The laxative effects of gallotannin-enriched extract isolated from Galla Rhois (GEGR) were investigated using a constipation model induced by loperamide (Lop) injection. After analysis for antioxidant activity of GEGR, alterations in the excretion parameters, histological structure, mucin secretion, and related protein levels were measured in the transverse colon of Sprague Dawley (SD) rats with Lop-induced constipation following treatment with 250, 500 and 1,000 mg/ml of GEGR. The number and weight of feces increased significantly by 48–79% and 128–159%, respectively, in the Lop+GEGR treated group relative to the Lop+vehicle treated group, while food intake and water consumption were maintained at a constant level. The thickness of mucosa, muscle and flat luminal surface, as well as the number of goblet cells and crypt of lieberkuhn were enhanced in the Lop+GEGR treated group. Moreover, mucin secretion increased significantly in a dose dependent manner in the Lop+GEGR treated group. Furthermore, the downstream signaling pathway of the muscarinic acetylcholine receptors (mAChR) M2 and M3 was recovered by GEGR treatment, although the expression level varied. The levels of Gα expression and inositol triphosphate (IP3) concentration were also recovered in the Lop+GEGR treated group relative to the Lop+vehicle treated group. The results of the present study provide strong evidence that tannins distributed in various medicinal plants are important candidates for improving chronic constipation induced by Lop treatment in animal models.

## 1. Introduction

Constipation is an acute or chronic gastrointestinal disease characterized by infrequent bowel movements, hard and dry feces, incomplete bowel evacuation and difficulty during defecation [1]. Although a variety of treatments are available, the best method for treatment of constipation is making simple changes to incorporate more fiber into the diet, drink plenty of fluids, and add exercise into the patient's daily routine. However, chemical drugs (laxatives) such as senna, correctol, exlax, senokot and gaviscon can also be prescribed help patients pass feces [2]. Most of these laxatives act as stimulants to increase bulkiness and soften stool, or as osmotic agents, enhancing water flow into the colon to promote elimination and trigger bowel movements [3].

Recently, several herbal plants and medicinal foods exhibiting laxative activity have received increased attention as novel therapeutic strategies for the treatment of constipation and its related diseases; however, more studies are required to support the pharmacological mechanism [4,5]. Several plant extracts containing tannins have also been reported to exhibit laxative activities based on their ability to increase feces number and weight. Leaf aqueous extract of *Mareya micrantha Mull*. *Arg*. containing flavonoids, alkaloids and tannins reduced loperamide induced constipation in a dose dependent manner [5], while the methanol extract of *Senna macranthera* containing flavonoids, tannins and coumarins showed laxative and intestinal motility effects in an animal model [6]. Moreover, increased production of feces was observed in animals treated with aqueous extracts of *Aloe ferox* Mill. and aqueous-methanol extract of *Fumaria parviflora* containing tannins [7,8]. Furthermore, *Urginea indica* Kunth. and *Phyllanthus emblica* containing alkaloids, tannins and coumarins induced an increased number and weight of feces in a dose dependent manner [9]. However, the laxative effects of Galla Rhois (GR, accounting for more than 98% of tannins) on the constipation induced by loperamide (Lop) injection have not yet been investigated in Sprague Dawley (SD) rats, even though gallotannin-enriched extract isolated from Galla Rhois (GEGR) has traditionally been used for treatment of diarrhea, several skin diseases, seminal emissions, excessive sweating, abscess, bleeding and chronic cough without any toxicity [10,11]. The use of GR to treat diarrhea has only been investigated based on an antibiotics activity in *Eimeria tenella* infected chickens and highly infectious post weaning piglets, regardless key role as laxatives for the regulation of bowel movement [12,13]. GR is the excrescence formed by parasitic aphids, primarily *Schlechtendalia chinensi*s Bell, on the leaf of sumac, *Rhus javanica* (Anacardiaceae). This compound has been found to have favorable ethnopharmacological properties [14]. In recent studies, GR exhibited good antibacterial activity against many pathogenic bacteria, including *Salmonella spp*. and *Escherichia coli* [15], as well as significant anti-inflammatory activity in lipopolysaccharide (LPS)-stimulated RAW264.7 macrophages [16]. GR was also found to exert anticancer activity against nasopharyngeal carcinoma cells [17] and to improve sensory motor function in a transient focal cerebral ischemia rat model [18].

Therefore, the present study was conducted to verify the laxative activity of GEGR containing high levels of tannins in a Lop-induced constipation model. Our results suggest that tannins can successfully induce laxative effects in the constipated animal model.

## 2. Materials and Methods

### 2.1. Preparation of GEGR

GEGR were prepared as described in previous studies [19,20]. Briefly, GR harvested in the Hongcheon area of Korea during October of 2013 was obtained from the Hongcheon National Agricultural Cooperation Federation (http://www.hcari.co.kr). Specimens were dried in a hot-air drying machine (JSR, Seoul, Korea) at 60°C and deposited as voucher specimens of GR roots (WPC-14-001) in the functional materials bank of the Wellbeing Regional Innovation System Center at Pusan National University (PNU). The aqueous extract of GR was obtained from powder of dry samples at 90°C for 9 h in a fixed liquor ratio (solid GR powder/water ratio, 1:10) using circulating extraction equipment (IKA Labortechnik, Staufen, Germany). These solutions were subsequently passed through a 0.4 μm filter, after which they were concentrated by vacuum evaporation and lyophilization using circulating extraction equipment (IKA Labortechnik, Staufen, Germany).

### 2.2. Analysis of functional compounds

The concentrations of gallic acid, methyl gallate and gallotanin in GEGR were measured as previously described [19,20]. Gallic acid monohydrate (IUPAC name; 3,4,5-trihydroxybenzoic acid, MW: 170.12 g/mol, Sigma-Aldrich Co., St. Louis, MO, USA), methyl gallate (IUPAC name; methyl 3,4,5-trihydroxybenzoate, MW: 184.15 g/mol, Sigma-Aldrich Co.) and gallotanin (IUPAC name; 3,5-dihydroxy-2-(3,4,5-trihydroxybenzoyl)oxy-6-[(3,4,5-trihydroxybenzoyl)oxymethyl]oxan-4-yl] 3,4,5-trihydroxybenzoate, MW: 1701.20 g/mol, Sigma-Aldrich Co.) were used as standard compounds during analysis of the main components of GEGR. The wavelengths of the maximum absorption of pure gallic acid, pure methyl gallate, commercial gallotannin, and gallnut extract were 212/257, 214/268, 213/278 and 212/275 nm, respectively. The UV-VIS spectra of pure gallic acid, pure methyl gallate, pure gallotannin, and the gallnut extract showed two bands at 212–214 nm and 257–278 nm, which were both assigned to the π→π* transitions of the given aromatic units and C = O groups in the UV-VIS region. Finally, the UV-Vis spectra were analyzed using a curve-resolving technique based on linear least-squares analysis to fit the combination of the Lorentzian and Gaussian curve shapes.

### 2.3. High performance liquid chromatography (HPLC) analysis

The HPLC system used for these experiments consisted of a Summit Dual-Gradient HPLC System (Dionex, USA) with a PDA UV-vis detector at the Korea Bio-IT Foundry Busan Center. Separation was carried out on an YMC-Triart C18 column (S-5 mm/12 nm, 150 mm × 4.6 mm I.D.) maintained at 40°C. The mobile phase consisted of solvent A (0.4% formic acid in water) and solvent B (acetonitrile). The gradient condition of the mobile phase was as follows: 0–5 min, 10% B; 5–6 min, 10–15% B; 6–40 min, 15% B; 40–41 min, 15–30% B; 41–50 min, 30% B; 50–55 min, 30–10% B; 55–60 min, 10% B. The injection volume was 5 ml in full loop injection. The flow rate was 0.8 ml/min and detection was performed at 280 nm.

### 2.4. Free radical scavenging activity

The scavenging activity of 2,2-diphenyl-1-picrylhydrazyl (DPPH) radical was measured as previously described [21]. Briefly, powdered extract was dissolved in 50% ethanol (EtOH,100 μL) solution containing 13 different concentrations of GEGRs (0.122 to 125 μg/ml) and mixed with 100 μL of 0.1 mM DPPH (Sigma-Aldrich Co.) in 95% EtOH solution or 100 μL of 95% EtOH solution, then incubated for 30 min at room temperature. Next, the absorbance of the reaction mixture was measured at 517 nm using a Versa-max plate reader (Molecular Devices, Sunnyvale, CA, USA). The DPPH radical scavenging activity of the GEGR was expressed as the percent decrease in absorbance relative to the control. The half maximal inhibitory concentration (IC)_50_ value is defined as the concentration of substrate that causes a 50% loss in DPPH activity.

### 2.5. Experimental design for animal study

The animal protocol used in this study was reviewed and approved based on the ethical procedures for scientific care set by the PNU-Institutional Animal Care and Use Committee (PNU-IACUC; Approval Number PNU-2014-0572). Adult SD rats purchased from Samtako Inc. (Osan, Korea) were handled in the PNU-Laboratory Animal Resources Center, which is accredited by the Korea Food and Drug Administration (FDA)(Accredited Unit Number-000231) and Association for Assessment and Accreditation of Laboratory Animal Care (AAALAC) International according to the National Institutes of Health guidelines (Accredited Unit Number; 001525). Animals were provided with *ad libitum* access to a standard irradiated chow diet (Samtako Inc.) consisting of moisture (12.5%), crude protein (25.43%), crude fat (6.06%), crude fiber (3.9%), crude ash (5.31%), calcium (1.14%) and phosphorus (0.99%) and water throughout the feeding study. During the experiment, these rats were maintained in a specific pathogen-free (SPF) state under a strict light cycle (lights on at 08:00 h and off at 20:00 h) at 23±2°C and 50±10% relative humidity without any environmental enrichments.

Constipation of SD rats was induced by Lop treatment as previously described [7,21]. First, 8-week-old SD rats (n = 42) were assigned to either a non-constipation group (n = 12) or a constipation group (n = 30). Constipation was induced by subcutaneous injection of Lop (4 mg/kg weight) in 0.9% sodium chloride twice a day for 3 days, whereas the non-constipation group was injected with 0.9% sodium chloride alone. The non-constipation group was further divided into a No treated group (No, n = 6) and a GEGR treated group (GEGR, n = 6). The No group was untreated during the experimental period, whereas the GEGR group received 1,000 mg/kg weight of GEGR at one time. Additionally, the constipation group was further divided into a Lop+vehicle treated group (Lop+vehicle, n = 6), Lop+low GEGR treated group (Lop+LoGEGR, n = 6), Lop+medium GEGR treated group (Lop+MiGEGR, n = 6), Lop+high GEGR treated group (Lop+HiGEGR, n = 6), and Lop+BisaC (Lop+BS, n = 6). Three Lop+GEGR treated groups received 250, 500 and 1,000 mg/kg body weight of GEGR, while the Lop+BS group received 3.3 mg/kg body weight of BS (Kolon Pharmaceuticals Inc., Gyenggido, Korea) once after the induction of constipation. The positive control (BS) consisted primarily of bisacodyl, docusate sodium and sennoside calcium. The physiological condition of all animals in each group was regularly monitored at 10 a.m. every day during the experimental periods, and no severely ill or dead animals were observed in this period. At 24 h after GEGR, BS and vehicle treatment, all animals were euthanized using CO_2_ gas and tissue samples were acquired and stored in Eppendorf tubes at -70°C until assay.

As detailed above, three doses of GEGR (250, 500 and 1,000 mg/kg body weight) were orally administered to SD rats (250 g) at one time. Human equivalent doses (HED) were calculated based on the body surface area using the following formula for dose translation [22,23]:

HED(mg/kg)≡Ratdose(mg/kg)×(RatKm/HumanKm)

where, the *Km* value for rats and humans is 6 and 37, respectively. Therefore, a 250, 500 or 1,000 mg single dose of GEGR in rats is equivalent to a 40.5 mg, 81 mg or 162 mg daily dose in humans, respectively. Further, the maximum daily dose (162 mg) of GEGR in humans is below 560 mg/kg body weight/day and 800 mg/kg body weight, which has been established as the total acceptable daily intake (ADI) of tannic acid and the no-effect level for food-grade tannic acid in rats [24].

### 2.6. Analysis of food intake, water intake and body weight

Alterations in food intake, water consumption and body weight of SD rats treated with Lop+GEGR were measured daily at 10:00 am throughout the experimental period using an electrical balance and a measuring cylinder. All measurements were performed three times to ensure accuracy.

### 2.7. Measurement of feces parameters

To collect pure feces and urine without any contamination, all SD rats were bred in metabolic cages (Daejong Instrument Industry Co., Ltd., Seoul, Korea) during the experimental period. The feces number and weight were measured as described in previous studies [4,7]. The feces excreted from each SD rat was collected at 10:00 am. Feces samples were weighed three times per sample using an electric balance, whereas the number of feces was counted three times.

### 2.8. RT-PCR

The frozen tissue of transverse colons was cut with scissors and rapidly homogenized in RNAzol B solution (Tet-Test Inc., Friendswood, TX, USA). Following isolation of the total RNA, its concentration was determined by UV-spectroscopy. The expression of mAChR M2 and M3 mRNA was subsequently examined by RT-PCR using 5 μg of the total RNA from transverse colons. Briefly, 500 ng of oligo-dT primer [ThermoFisher Scientific Inc., Waltham, MA, USA] was annealed with the template RNA at 70°C for 10 min. The complementary DNA, which served as the template for subsequent amplification, was then synthesized by adding dATP, dCTP, dGTP and dTTP with 200 units of reverse transcriptase and 10 pmole of sense and antisense primers. Next, amplification was conducted by subjecting the samples to 28 cycles of 30 sec at 94°C, 30 sec at 62°C and 45 sec at 72°C in a Perkin-Elmer Thermal Cycler. In each case, negative-RT controls were included to differentiate the DNA and RNA products. RT-PCR was also performed using primers specific to β-actin to ensure the RNA integrity. The primer sequences used to evaluate mAChR M2 expression were as follows: sense primer, 5’-CCAGT ATCTC CAAGT CTGGT GCAAG G-3’, antisense primer, 5’-GTTCT TGTAA CACAT GAGGA GGTGC-3’. The primer sequences used to evaluate mAChR M3 expression were as follows: sense primer, 5’-GTCAC TTCTG GTTCA CCACC AAGAG C-3’, antisense primer, 5’-GTGTT CACCA GGACC ATGAT GTTGT AGG-3’. The sequences of the β-actin sense and antisense primers were 5’-TGGAA TCCTG TGGCA TCCAT GAAAC-3’ and 5’-TAAAA CGCAG CTCAG TAACA GTCCG-3’, respectively. The level of the PCR products was quantified using a Kodak Electrophoresis Documentation and Analysis System 120 and 1% agarose gels.

### 2.9. Western blotting

Total proteins collected from the transverse colons of subset groups (No, GEGR, Lop+vehicle, Lop+LoGEGR, Lop+MeGEGR, Lop+HiGEGR and Lop+BS treated SD rats) were separated by 4%–20% sodium dodecyl sulfate-polyacrylamide gel electrophoresis (SDS-PAGE) for 3 h, after which the resolved proteins were transferred to nitrocellulose membranes for 2 h at 40 V. Each membrane was then incubated separately with primary antibody, anti-mAChR M2 antibody (Alomone Labs, Jerusalem, Israel), anti-PI-3K (Cell Signaling Technology Inc., Cambridge, MA, USA), anti-p-PI3K (Cell Signaling Technology Inc., Cambridge, MA, USA), anti-mAChR M3 antibody (Alomone Labs, Jerusalem, Israel), anti-PKC (Cell Signaling Technology Inc.), anti-p-PKC (Cell Signaling Technology Inc.), anti-Gα (Abcame, Cambridge, UK) or anti-actin (Sigma-Aldrich Co.) overnight at 4°C. Next, the membranes were washed with washing buffer (137 mM NaCl, 2.7 mM KCl, 10 mM Na_2_HPO_4_, 2 mM KH_2_PO_4_, and 0.05% Tween 20) and incubated with horseradish peroxidase-conjugated goat anti-rabbit IgG (Zymed Laboratories, South San Francisco, CA, USA) at a dilution of 1:1,000 and room temperature for 2 h. Finally, the membrane blots were developed using Chemiluminescence Reagent Plus kits (Pfizer, New York, NY, USA and Pharmacia, New York, NY, USA).

### 2.10. Histopathological analysis

Transverse colons collected from subset constipation groups treated with vehicle, GEGR and BS were fixed with 10% formalin for 12 h, embedded in paraffin wax, and then sectioned into 5 μm thick slices that were stained with hematoxylin and eosin (H&E, Sigma-Aldrich Co.). Morphological features of these sections were observed under light microscopy, after which the mucosa thickness, muscle thickness, flat luminal surface thickness, number of goblet cells and number of crypts of lieberkuhn were measured using Leica Application Suite (Leica Microsystems, Switzerland).

For mucin staining, transverse colons collected from SD rats that had been cotreated with Lop+GEGR were fixed with 10% formalin for 48 h, embedded in paraffin wax, and then sectioned into 3 μm thick slices that were subsequently deparaffinized with xylene and rehydrated. Next, the tissue sections on the slides were rinsed with distilled water and stained using an Alcian Blue Stain kit (IHC WORLD, Woodstock, MD, USA). Finally, the morphological features in the stained colon sections were observed by light microscopy.

### 2.11. Measurement of IP3 concentration

The levels of IP3 were determined using an IP3 ELISA kit (Cusabio Biotech Co., Ltd., Wuhan, China) according to the manufacturer’s instructions. Briefly, the transverse colons (100 mg) were washed and homogenized in ice-cold PBS (pH 7.2–7.4) with a glass homogenizer (Sigma-Aldrich Co.). The tissue lysates were then centrifuged at 1,000 rpm for 5 min at room temperature, after which the supernatant was collected for analysis. An anti-IP3 detection antibody was added and incubated at 37°C for 60 min, after which substrate solution was added and incubated for 15 min at 37°C. The reaction was terminated following the addition of stop solution and the plates were read at an absorbance of 450 nm using a Molecular Devices VERSA max Plate reader (Sunnyvale, CA, USA).

### 2.12. Statistical analysis

One-way ANOVA (SPSS for Windows, Release 10.10, Standard Version, Chicago, IL, USA) was used to determine the variance and identify significant differences between the non-constipation group and constipation group, as well as between the vehicle treated group and the GEGR/BS treated group within the constipation group. All values are presented as the means ± standard deviation (SD). A *P* <0.05 was considered significant.

## 3. Results

### 3.1. Main components of GEGR

**Fig 1:**
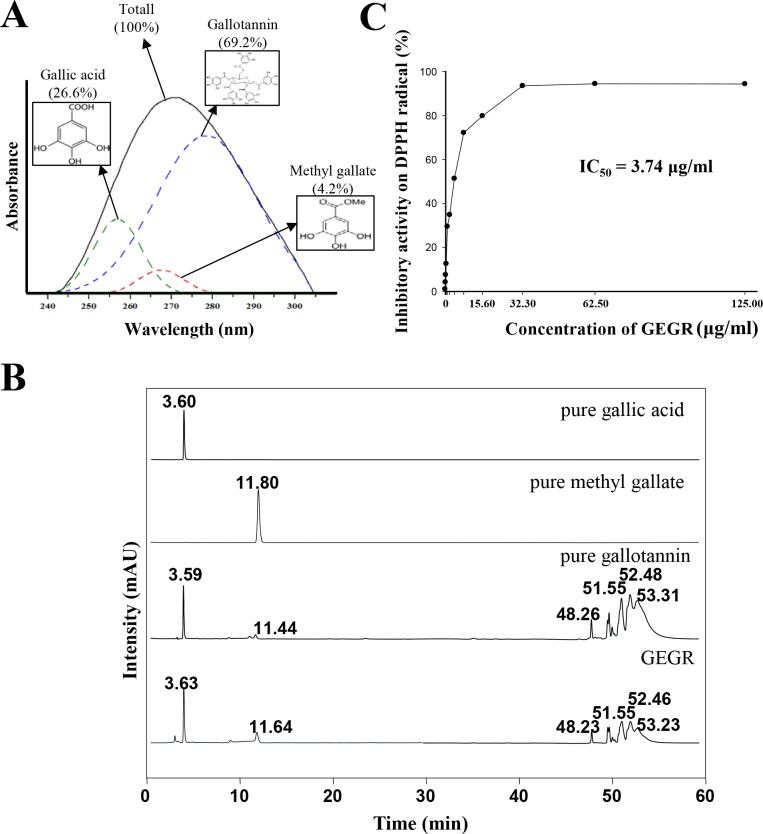
Concentration of major components and free radical scavenging activity of GEGR. (A) The distribution of three major components of GEGR, gallotannin, gallic acid and methylgallate, was analyzed based on their UV-Vis spectra. (B) HPLC chromatograms of pure gallic acid (commercial chemical), pure methylgallate (commercial chemical), pure gallotannin (commercial chemical) and GR extract. (C) DPPH radical scavenging activity of GEGR. The data shown represent the means ± SD of three replicates.

In a previous study [19], the levels of gallic acid, methyl gallate and gallotannin were measured at 257 nm, 268 nm and 278 nm, respectively. Gallotannin underwent a reaction with glucose, and dimers or higher oligomers of gallic acid comprised 69.2% of the GEGR, while two other compounds with a similar chemical structure (gallic acid and methyl gallate) accounted for 26.6% and 4.2% of the same sample, respectively (Fig 1A). In addition, HPLC curves of GEGR revealed characteristic peaks for gallic acid (3.58 min), methyl gallate (11.4 min), and gallotannin (48.26, 51.55, 52.46, and 53.23 min) (Fig 1B). Moreover, the inhibitory activity against DPPH radical was rapidly increased by the addition of 0.12–32.30 μg/ml of GEGR. Based on these data, the IC_50_ value of GEGR was determined to be 3.75 μg/ml (Fig 1C). Taken together, these results indicate that GEGR contained three major components, gallic acid, methyl gallate and gallotannin, and had very strong DPPH radical scavenging activity.

### 3.2. Effects of GEGR treatment on feeding behavior and feces parameters

We investigated whether GEGR treatment could affect the feeding behavior and feces parameters of constipated rats. To achieve this, the food intake, water consumption, and feces number and weight were measured in constipated SD rats after GEGR treatment. As shown Table 1, three factors related to feeding behavior, body weight, food intake and water consumption, did not differ significantly among groups. However, the decline in feces number and weight in the Lop+ vehicle treated group was almost recovered in the Lop+MiGEGR, Lop+HiGEGR and Lop+BS treated groups compared to those in the No and GEGR treated group, while the Lop+LoGEGR treated group did not recover (Table 1). Therefore, the above results suggest that GEGR treatment could improve Lop-induced constipation in SD rats through stimulation of feces excretion without alteration of feeding behavior. However, these laxative effects can be induced by treatment with more than 500 mg/kg of GEGR.

**Table 1:** Alteration of feeding behavior and constipation parameters in the constipated SD rats after GEGR treatment.

Category	No	GEGR	Loperamide				
			Vehicle	LoGEGR	MiGEGR	HiGEGR	BS
Body weight (g)	288±2.9	292±2.2	293±4.4	287±2.0	287±3.4	292±2.9	289±2.3
Food intake (g/day)	26.9±1.8	28.2±2.3	27.6±3.1	25.1±2.5	26.5±2.8	27.3±2.1	25.2±1.7
Water consumption (ml)	25.1±2.0	26.2±2.1	26.4±2.3	24.6±1.8	23.9±3.1	25.1±2.6	27.0±1.7
Feces number (ea)	40.3±1.5	42.8±1.9	29.7±1.7^a^	33.5±2.8^a^	43.0±2.0^b^	52.3±1.2^b^	50.8±2.3^b^
Feces weight (g)	7.7±0.3	8.0±0.4	3.2±0.2^a^	4.0±0.5^a^^,^^b^	7.3±0.2^b^	8.3±0.2^b^	8.6±0.3^b^

a, *P*<0.05 indicates significance when compared with the non-constipation group.

b, *P*<0.05 indicates significance when compared with the Lop+vehicle treated group.

### 3.3. Effect of GEGR on histological alterations of the transverse colon

**Fig 2:**
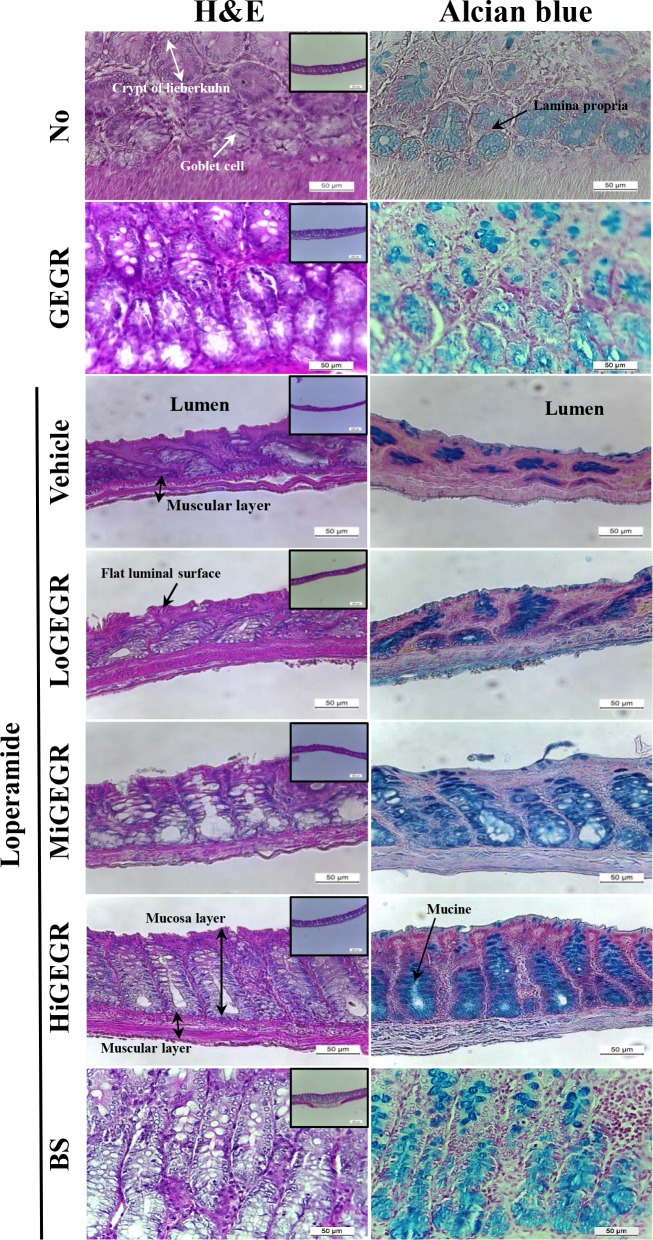
Alteration of histological structure in Lop-induced constipated rats. H&E stained sections of transverse colon rats from the No treated group, GEGR treated group, Lop+vehicle treated group, Lop+GEGR treated group or Lop+BS treated group were observed at 100× (upper corner in left column) and 200× (left column) using a light microscope. Mucin secreted from crypt layer cells was stained with alcian blue at pH 2.5, and their structures were observed at 200× (right column). Five to six rats per group were assayed in triplicate by H&E and alcian blue staining.

To investigate whether GEGR treatment could induce alterations of the histological structure of the transverse colon, alterations in several histological parameters were measured in H&E stained transverse colons of SD rats treated with GEGR. Although histopathological changes were observed in all Lop+GEGR treated groups, the statistical significance varied in each group. The thickness of mucosa, muscle and flat luminal surface was significantly shorter in the Lop+vehicle treated group than the No treated group and the GEGR treated group (Fig 2). However, these levels rapidly increased by 122.6%, 25.1% and 82.6% following Lop+HiGEGR cotreatment when compared with the Lop+vehicle treated group (Table 2). Conversely, the Lop+MiGEGR treated group only showed an increase in mucosa thickness, while consistent levels of muscle thickness and a flat luminal surface thickness were maintained. Additionally, the Lop+LoGEGR treated group did not show any significant alterations of these levels (Fig 2). Furthermore, the number of goblet cells and crypt of lieberkuhn were 41% and 55% lower in the Lop+vehicle treated group than the No treated group, respectively (Table 2). However, these levels were enhanced in the Lop+MiGEGR (43 and 69%) and Lop+HiGEGR (948% and 96%) treated groups, while they were maintained at a constant level in the Lop+LoGEGR treated group (Table 2). Moreover, the histological alterations of transverse colon in the Lop+BS treated group as a positive control were completely recovered to those of the No and GEGR treated group (Fig 2 and Table 2). Therefore, the aforementioned results indicate that treatment with high doses of GEGR may induce recovery of the histopathological structure of the transverse colon in the Lop-induced constipation model.

**Table 2:** Histopathological alteration of the constipated SD rats after GEGR treatment.

Category	No	GEGR	Loperamide				
			Vehicle	LoGEGR	MiGEGR	HiGEGR	BS
Mucosa thickness (μm)	496.4±3.8	487.6±2.3	146.3±2.1^a^	152.5±1.7^a^	245.6±1.8^a^^,^^b^	325.2±2.3^a^^,^^b^	458.8±1.5^b^
Muscle thickness (μm)	95.1±3.3	92.2±2.4	68.8±2.6^a^	78.3±2.1^a^	71.2±1.3^a^^,^^b^	85.6±1.0^a^^,^^b^	94.1±1.6^b^
Flat luminal surface thickness (μm)	58.8±2.4	59.9±1.8	23.5±1.1^a^	22.9±2.6^a^	29.8±1.5^a^^,^^b^	42.3±2.4^a^^,^^b^	48.5±2.2^a^^,^^b^
Number of goblet cell (ea)	457.3±21.4	468.1±30.2	271.7±18.3^a^	299.4±25.8^a^	388.1±35.1^a^^,^^b^	402.1±30.8^a^^,^^b^	486.7±31.9^b^
Number of crypt of lieberkuhn (ea)	51.1±1.8	53.3±0.5	23.5±2.0^a^	24.8±1.2^a^	39.4±2.3^a^^,^^b^	45.2±1.8^a^^,^^b^	49.8±1.1^b^

a, *P*<0.05 indicates significance when compared with the non-constipation group.

b, *P*<0.05 indicates significance when compared with the Lop+vehicle treated constipation group.

### 3.4. Effect of GEGR on the mucin secretion capability

To investigate the effects of GEGR treatment on the ability to secrete mucin, the levels of mucin in tissue sections stained with Alcian blue were observed in the transverse colon of the subset group. The total level of mucin increased dramatically in the Lop+GEGR treated group compared to the Lop+vehicle treated group, although the highest level was detected in the Lop+HiGEGR treated group. The region stained with dark blue was concentrated into the crypt in the mucosa layer of the transverse colon. Furthermore, a similar level of mucin secretion was observed in both the GEGR and Lop+BS treated group (Fig 2, right column). These results show that the administration of high concentrations of GEGR may enhance the ability to secrete mucin in the transverse colon of constipated SD rats.

### 3.5. Effects of GEGR treatment on the mAchRs downstream signaling pathway

**Fig 3:**
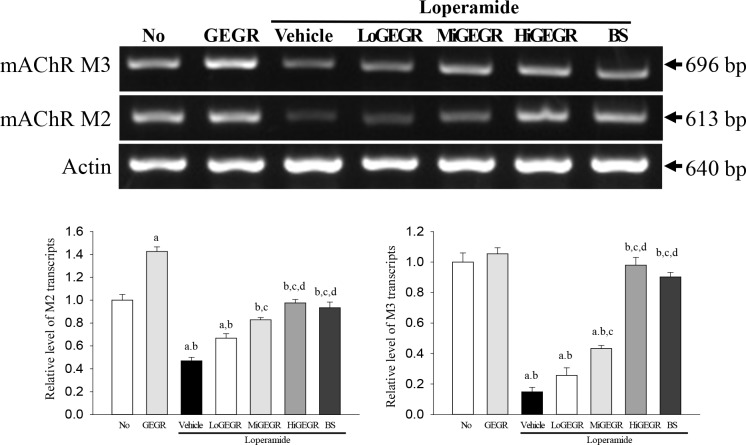
Expression of mAChRs transcript in the transverse colon. The levels of mAChR M2 and M3 transcripts in the total mRNA of transverse colons were measured by RT-PCR using specific primers. After the intensity of each band was determined using an imaging densitometer, the relative levels of mAChR M2 and M3 transcripts were calculated based on the intensity of actin transcripts. Five to six rats per group were assayed in triplicate by RT-PCR assays. Data represent the means±SD of three replicates. a, p<0.05 compared to the No treated group. b, p<0.05 compared to the Lop+vehicle treated group.

**Fig 4:**
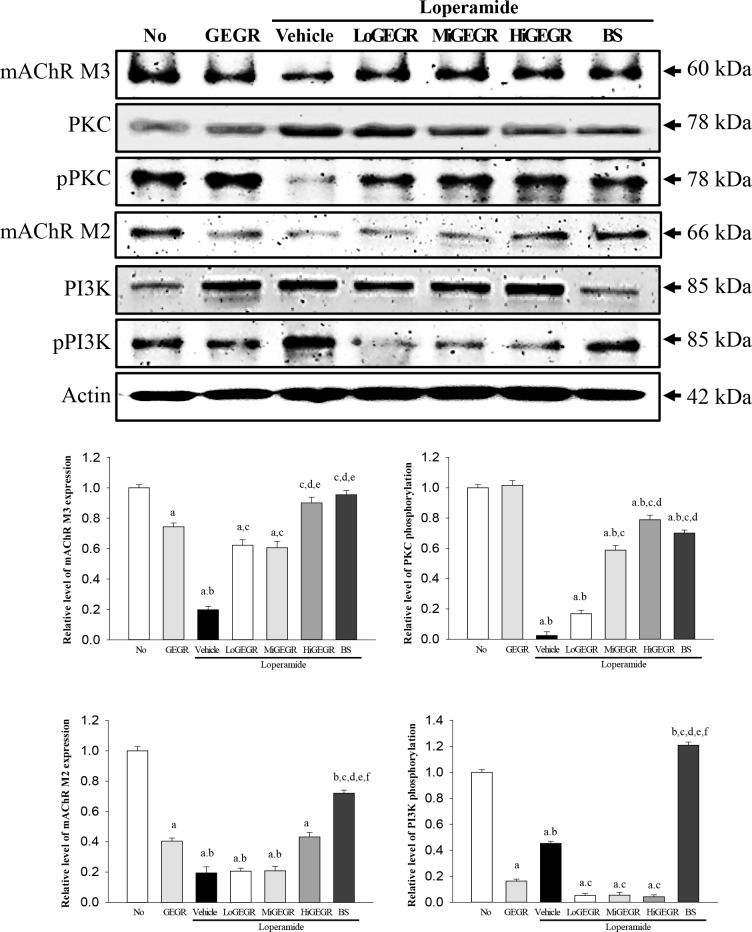
Expression of key proteins in the mAChR M2 and M3 signaling pathway. The expression of several related proteins in the mAChR M2 and M3 signaling pathway was measured by Western blot analysis using HRP-labeled anti-rabbit IgG antibody. After the intensity of each band was determined using an imaging densitometer, the relative levels of six proteins were calculated based on the intensity of actin protein. Five to six rats per group were assayed in triplicate by Western blotting. Data represent the means ± SD of three replicates. a, p<0.05 compared to the No treated group. b, p<0.05 compared to the Lop+vehicle treated group.

To investigate whether the laxative effects of GEGR were accompanied by alterations in the mAChRs downstream signaling, the expression of the related mRNA and proteins was measured in the transverse colon of the Lop+GEGR treated group. A similar pattern was detected in the expression of the two mAchRs mRNA in all experimental groups. The expression level of the mRNA of the two receptors was lower in the Lop+vehicle treated group than the No and GEGR treated group. However, their levels gradually increased in all Lop+GEGR treated groups, as well as in the Lop+BS treated group (Fig 3). Additionally, the expression patterns of these proteins primarily reflected the expression level of mAChR mRNA. The expression levels of mAChR M3 proteins were increased in all Lop+GEGR and Lop+BS treated groups, while those of mAChR M2 proteins were only enhanced in the Lop+HiGEGR and Lop+BS treated group (Fig 4). Moreover, a different response was detected in the phosphorylation levels of PKC and PI3K, members of the downstream pathway of mAChR M2 and M3. The phosphorylation levels of these proteins were recovered in the GEGR treated group compared with the Lop+vehicle treated group, even if the Lop+vehicle treated group showed lower or higher levels than the No and GEGR treated group (Fig 4). Therefore, the results of the present study indicate that the laxative effects of GEGR may correlate with the regulation of mAChRs expression at the transcriptional and translational level, as well as their downstream signals in the transverse colons of constipated SD rats.

### 3.6. Effect of GEGR treatment on G protein signaling

**Fig 5:**
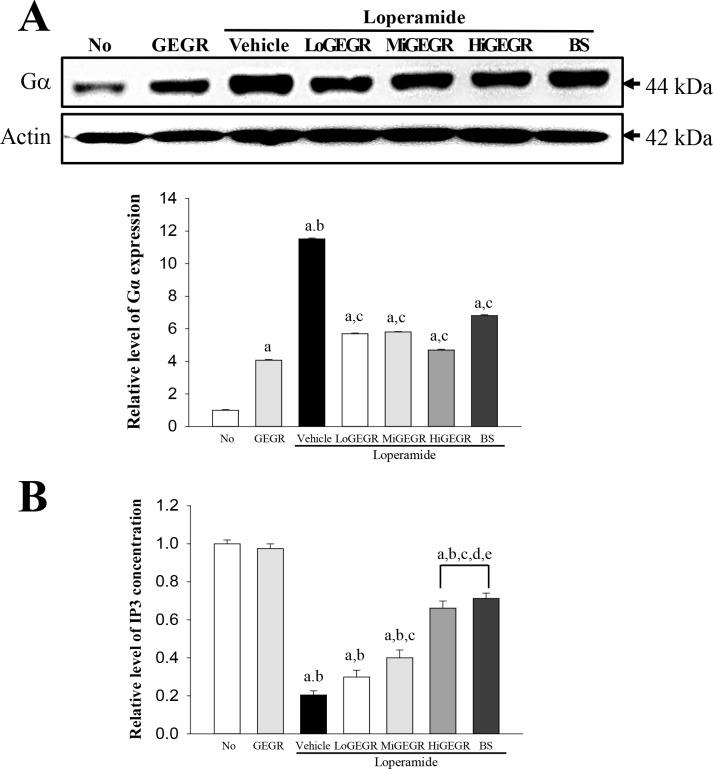
Alteration of the expression of Gα proteins and IP3 concentration. (A) The expression of Gα within G protein signaling was measured by Western blotting using HRP-labeled anti-rabbit IgG antibody. After the intensity of each band was determined using an imaging densitometer, the relative levels of protein were calculated based on the intensity of actin protein. (B) The tissue homogenate used to measure the IP3 concentration was prepared from transverse colons collected from SD rats of subset groups. The IP3 concentration was quantified by an enzyme-linked immunosorbent assay with a sensitivity of 0.39 ng/ml and an inter-assay coefficient of variation of 1.56–100 ng/ml. Data shown are the means ± SD (n = 5). a, *p*<0.05 compared to the No treated group. b, *p*<0.05 compared to the Lop+vehicle treated group.

Finally, we examined whether mAChR alteration induced by GEGR treatment was accompanied by altered G protein signaling by measuring the expression of Gα protein and the concentration of IP3 in the transverse colon of subset groups. Gα expression was higher in the Lop+vehicle treated group than the No and GEGR treated group. Following Lop and GEGR cotreatment, this level was significantly recovered relative to that of the No treated group without the effect of concentration increase (Fig 5A). However, IP3 concentration showed the opposite pattern of Gα expression. Specifically, these values decreased by 81% in Lop+vehicle relative to the No treated group, but IP3 concentration was gradually increased in all Lop+GEGR treated groups in a dose dependent manner (Fig 5B). The above results provide the first evidence that treatment with three different concentrations of GEGR may be correlated with the recovery of Gα expression and IP3 concentration in the Lop-induced constipation model.

## 4. Discussion

Many studies have investigated natural products to develop therapeutic drugs for the treatment of gastrointestinal diseases. In an effort to identify candidate drugs for the treatment of chronic constipation and verify the role of tannins as key laxatives, we investigated the laxative effects of GEGR in Lop-induced constipated rats. Our results demonstrated that GEGR treatment is associated with the recovery of structure and function in the transversal colon of constipated SD rats. Moreover, tannins can be considered a laxative for treatment of humans.

Tannins are abundant secondary metabolites that comprise a large, diverse group of polyphenolic polymers with excellent antioxidant activity found in several species of plants [25,26]. To date, many studies have reported the therapeutic value of tannins in various chronic diseases. For example, they appear to decrease blood pressure, reduce the risk of cancer, stimulate the immune system, improve diabetes, facilitate burn wound healing, and have anti-bacterial activity [27,28]. They also exert beneficial effects on colitis through suppression of inflammation, attenuation of colonic injury and modulation of pathogenic bacteria [25,29]. Several herbal plants containing tannins such as *Mareya micrantha*, *Aloe ferox* Mill., *Fumaria parviflora* Linn., and *Urginea indica* Kunth. have also been shown to alleviate the chronic symptoms of constipation [5,7–9]. Based on these results, we hypothesized that GEGR would exert laxative effects on the constipation induced by Lop treatment in SD rats. Specifically, we investigated whether oral administration of GEGR for 24 h improves the symptoms of constipation via regulation of excretion parameters, histological structure, mucin secretion and mAchRs downstream signaling.

In most constipation related studies, one of the key parameters is a significant decrease in feces excretion in chemical- or diet-induced animal models. Several feces-related factors including feces number, weight and water content were shown to be dramatically decreased in Lop-injected rats [4,14]. However, these changes were rapidly recovered by treatment with herbal medicines such as *Aloe ferox Mill*., *Liriope platyphylla* and *Mareya micrantha Mull*. [5,7,21]. As shown in Table 1, the number and weight of feces was also enhanced by 45–76% and 128–159%, respectively, in the Lop+GEGR treated group relative to the Lop+vehicle treated group. Similar results were observed in previous studies investigating the laxative effects of tannin-containing extracts. For example, the feces weight increased by 176–272% in rats treated with the aqueous extract of *Mareya micrantha*, while it was enhanced by 71–143% in the case of *Aloe ferox* Mill. [5,7]. Additionally, the aqueous-methanol extract of *Fumaria parviflora* and aqueous extract of *L*. *platyphylla* (AEtLP) induced an increase of 52–80% and 86% in the number of feces [8,21].

Although histological data has been widely employed to demonstrate significant alterations in the intestines of constipated animals, investigations of the laxative effects of tannin containing extracts did not reveal alterations in histological structure. However, other herbal medicines with laxative effects effectively induced the recovery of histological structure in the intestines of animals. The thickness of the distal colon and area of crypt epithelial cells were enhanced by treatment with *Ficus carica paste*, while *L*. *platyphylla* treatment induced an increase in the villus length, intestinal gland thickness and muscle thickness [4,21]. In the present study, a similar effect on the histological structure was observed in the Lop+GEGR treated group (Fig 2). This study provides the first detailed information regarding the ability of GEGR to induce histological changes in mucosa, muscle, and flat luminal surface thickness, number of goblet cells, and number of crypts of lieberkuhn in constipated rats.

The colorectal mucosa is effectively protected by mucin from a variety of types of damage induced by mechanical and chemical factors [30]. The level of secreted mucin was shown to decrease significantly upon Lop treatment [31]. Additionally, their levels were recovered in response to treatment with several compounds or herbal extracts such as carrageenan, chondroitin and *L*. *platyphylla* [21,31]. Similar effects on mucin secretion were detected in the present study. As shown in Fig 2, the total level of mucin decreased in the Lop+vehicle treated group, but was recovered in the Lop+GEGR treated group. Therefore, these results suggest that the regulation of mucin secretion is an important mechanism to improve constipation in Lop-induced animal models.

The involvement of mAChR M2 and M3 in the laxative effects of herbal medicine was first reported in the transverse colon of SD rats treated with AEtLP [21]. The level of mAChR M2 and M3 mRNA was higher in the transverse colon of constipated rats than the No treated group. Following treatment with AEtLP, these levels decreased, although they did not completely recover to those of the No treated group in any of the treatment groups. The expression of PI3K and PKC protein was also very similar to the level of mAChR M2 and M3 mRNA in a Lop+AEtLP treated group [21]. Overall, the results of the present study provide the first evidence that the level of mAChR M2 and M3 expression and the down-stream signaling pathway can be recovered by GEGR treatment. Furthermore, our results showed that the laxative effects of GEGR may be correlated with the expression of Gα protein and IP3 concentration. Gα protein and IP3 are key regulators of the G-protein coupled receptor downstream signaling pathway, which is activated by peptide hormones, neurotransmitters and odor molecules [32]. Moreover, a specific type of G protein-coupled receptor was associated with G-protein gated ion channels, which regulate the flow of potassium, calcium, sodium and chloride across the plasma membrane [32]. Among these channels, the calcium channel and function of clamodulin was blocked or inhibited by non-opioid effects of Lop [33], while Lop acts as an opioid receptor agonist for the mu opioid receptors in the myenteric plexus large intestines and is therefore used to treat diarrhea caused by gastroenteritis or inflammatory bowel disease [34].

The microorganisms distributed in the intestine have been shown to contribute to production of various disease conditions by biotransforming a variety of endogenous or ingested compounds to harmful agents or by inhibiting the generation of beneficial products [15]. Indeed, the number of clostridia and bifidobacteria was significantly higher in children with constipation, while the total population of lactobacilli and bifidobacteria was reduced among adult patients [35,36]. Moreover, the injection of vancomycin into constipation patients induced an increase in stool volume, frequency, consistency and ease of defecation [37]. Many studies have focused on the identification and evaluation of herbal medicine with antibacterial effects to develop novel drugs for constipation. Additionally, GR has been considered a potential candidate with antibacterial activity among various Oriental medicinal plants. The growth-inhibitory effects of GR and various active components on intestinal bacteria were characterized in several studies. The methanol extract of GR showed growth-inhibitory activity toward both *Clostridium perfringens* and *Escherichia coli* [38], as well as therapeutic effects toward *Streptococcus suis* infection in BALB/c mice [39]. Moreover, methyl gallate and gallic acid at 10 mg disc^-1^ clearly inhibited intestinal bacteria, including *Clostridium* p*erfringens*, *Clostridium paraputrificum*, *Eubacterium limosum*, *Bacteroides fagilis*, *Staphylococus aureus* and *Escherichia coli* [15]. Therefore, it is possible that the laxative effects of GR observed in our study are associated with antibacterial activity. However, more studies are needed to understand how GR affects the distribution of intestinal microorganisms, although our results may have been focused on the correlation between neuronal regulation and the effects of GEGR.

## 5. Conclusion

Taken together, the results of the present study indicate that GEGR could induce the recovery of excretion parameters, histological structure and mucin secretion, while recovering mAChRs and G protein signaling. In addition, these results successfully demonstrated that tannins may contribute to the relief of constipation in Lop-induced constipated SD rats. Furthermore, our results show the possibility that GEGR has the potential for application to medical therapy because of its lower cost, increased safety and easier manufacturing process when compared with commercial chemical drugs. However, it should be noted that the present study was limited in that it used only one type of animal model for constipation and animals were only examined for up to 24 h. Additional long term studies and model trials are necessary to clarify the laxative effects of GEGR and utilize this material to improve human health.
